# Predictive values and other quality criteria of the German version of the Nurse-Work Instability Scale (Nurse-WIS) – follow-up survey findings of a prospective study of a cohort of geriatric care workers

**DOI:** 10.1186/s12995-014-0030-9

**Published:** 2014-09-11

**Authors:** Melanie Harling, Anja Schablon, Claudia Peters, Albert Nienhaus

**Affiliations:** 1 Competence Centre for Epidemiology and Health Services Research in Nursing (CVcare), University Medical Center Hamburg-Eppendorf, Martinistr. 52, Building O17, Hamburg, 20246, Germany; 2 Institution for Statutory Accident Insurance and Prevention in the Health and Welfare Services, Department of Occupational Health Research, Pappelallee 35/37, Hamburg, 22089, Germany

**Keywords:** Nurse-work instability scale, Nurses, Musculoskeletal disorders, Long-term sick leave

## Abstract

**Background:**

Until now there has been a lack of effective screening instruments for health care workers at risk. To counteract the forecast shortage for health care workers, the offer of early interventions to maintain their work ability will become a central concern. The Nurse-Work Instability Scale (Nurse-WIS) seems to be suitable as a screening instrument and therefore a prospective study of a cohort of nursing staff from nursing homes was undertaken to validate the Nurse-Work Instability Scale (Nurse-WIS).

**Methods:**

The follow-up data was used to test the sensitivity, specificity and the predictive values of the Nurse-WIS. The participants answered a questionnaire in the baseline investigation (T1) and in a follow-up 12 month after baseline. The hypothesis was that geriatric care workers with an increased risk according to the Nurse-WIS in T1 would be more likely to have taken long-term sick leave or drawn a pension for reduced work capacity in T2.

**Results:**

396 persons took part in T1 (21.3% response), 225 in T2 (42.3% loss-to-follow-up). In T1, 28.4% indicated an increased risk according to the Nurse-WIS. In T2, 10.2% had taken long-term sick leave or had drawn a pension for reduced work capacity. The sensitivity is 73.9% (95%-CI 55.7%–92.3%), the specificity is 76.7% (95%-CI 71.2%–82.8%). The ROC AUC indicated a moderate precision for the scale, at 0.74 (95%-CI 0.64–0.84).

The PPV of the Nurse-WIS is 26.6%, and the NPV is 96.3%. For those with an increased risk according to the Nurse-WIS, the probability in T2 of long-term sick leave or a pension for reduced work capacity is around eight times higher (OR 8.3, 95%-CI 2.90–23.07). Persons who had indicated a long-term sick leave or made an application for a pension for reduced work capacity in T1 had a 17 times higher risk (OR 17.4, 95%-CI 3.34–90.55).

**Conclusion:**

The German version of the Nurse-WIS appears to be a valid instrument with satisfactory predictive capabilities for recording an impending long-term sick leave. Whether the Nurse-WIS can be used as a screening tool which helps to design risk adjusted prevention programs for the afflicted nurse should be studied.

## 1 Introduction

As in other countries, the number of people in need of care is increasingly noticeably in Germany, and in order to ensure that they are provided for, an increase in the demand for health care workers is anticipated – a demand that can hardly be satisfied [1],[2].

Nursing is among the high-risk occupations as regards work-related back pain, in particular low back problems, with a point prevalence of approximately 17%, an annual prevalence of 40–50% and a lifetime prevalence of 35–80% [3]-[8]. Furthermore, the prevalence of presenteeism due to low back pain (58%) was found to be very high among registered nurses [9], and presenteeism was related to future sick leave [10]. Disorders of the musculoskeletal system are also the most frequent reason for long-term sick leave in Germany [11]. At the same time, burnout and psychological impairments are common among health care workers [12]-[16].

Harling et al. [17] established that health care workers are more likely to undergo rehabilitation due to musculoskeletal disorders than other occupational groups and that the risk of a pension due to reduced work capacity was higher following rehabilitation. In addition, health care workers were more likely to draw a pension for reduced work capacity than all other occupational groups.

Because of these mutually dependent factors, the focus is shifting to maintaining the work capacity of health care workers. The literature has shown that multifactor interventions based on a risk assessment programme are most likely to be successful [18]. Interventions targeted at persons with initial symptoms of a musculoskeletal disorder [19]-[21] or persons with an increased risk of a reduced work capacity [22] were also effective. But until now, effective screening instruments for the purpose of offering early interventions for health care workers at risk are missing. The Nurse-Work Instability Scale (Nurse-WIS) is a new questionnaire that seems to meet these requirements [23]. The aim of our study was to create a German Version of the Nurse-WIS and to validate this version in a prospective study of a cohort of nursing staff from nursing homes. The results of the baseline investigation were already published by Harling et al. [24]. The findings of the follow-up survey to test the sensitivity, specificity and predictive values of the Nurse-WIS are presented below. The hypothesis is that geriatric care workers who showed an increased risk according to the Nurse-WIS in the baseline survey will be more likely to have taken long-term sick leave in the follow-up.

## 2 Methods

### 2.1 The Nurse-Work Instability Scale (Nurse-WIS)

Work instability describes a discrepancy between the demands that the occupation places on the person and the person’s individual capabilities. Interventions at this point in time, which resolve this discrepancy, may prevent long-term sick leave and reduced capacity to work. This concept has previously been explored in relation to various clinical specialties [23]-[27].

The Nurse-WIS is an occupation-specific instrument for health care workers. In addition to the problems of a musculoskeletal disorder, it includes psychosocial factors. In the original English version, the scale consists of 30 items that can be answered with 1 = true and 0 = false. The points for all answers are added to give a total score. The higher the total score, the higher the risk of work instability (<10 points = low risk, 10–19 points = moderate risk, ≥ 20 points = increased risk) [23].

At the start of the study, the scale was translated into German in a forward-backward procedure. In the German version, the scale consists of 28 items because according to the item discrimination two items were unsuitable and according to Cronbach’s alpha, good measuring accuracy is achieved for the scale of 28 items. More detailed information about the Nurse-WIS and the translation of the Nurse-WIS into German is published by Harling et al. [24].

### 2.2 Study design and study participants

In order to validate the Nurse-WIS, a prospective study of a cohort of geriatric care workers was conducted over a period of 12 months. The cohort was studied in two survey periods. T1, the baseline survey, was conducted between September and December 2010. T2 took place twelve months later (September 2011- January 2012). Each participant was sent a second questionnaire that, along with other information, primarily collected data on sick leave. More information about the study design, recruitment of participants and the findings of the data from T1 is published by Harling et al. [24].

The study was conducted following the requirements of the Helsinki Declaration. The ethics commission of the Hamburg Medical Association also gave a positive verdict on the conduct of the study (reference number PV3463).

### 2.3 The survey instrument and criteria for inclusion and exclusion

For T2, a modified version of the survey instrument for T1 was used (the precise structure of the questionnaire can be seen at Harling et al. [24]). Health care workers who were no longer professionally active because of pregnancy, parental leave or unemployment were excluded from the analysis. Also excluded were voluntary assistants and health care workers who had not provided any information about sick leave. Participants receiving pension at T1 were also excluded.

### 2.4 Definition of the outcome variable

The central test variables for T2 are ‘Number of days’ sick leave and the reason for each case of absence in the previous 12 months’ and ‘Details of any application for early retirement or for a pension for reduced work capacity’. In Germany, long-term sick leave is an absence of > 42 days, since after this period continued payment of salary by the employer is replaced by sick pay from the statutory health insurance provider [11]. A pension for reduced work capacity is payable if, because of a considerable impairment to health, employment is no longer possible, or only to a limited extent. Since the Nurse-WIS is meant to determine the risk of long-term sick leave or a pension for reduced work capacity, an outcome variable was defined on the basis of these data. As described below, a dichotomous outcome variable was defined that includes long-term sick leave because of musculoskeletal disorders (MSD) and psychological impairments to well-being, as well as pensions for reduced work capacity:

 ➢ **Long-term sick leave (>42 days) due to work-related musculoskeletal disorders (MSD)**

Since the Nurse-WIS is not suitable for predicting long-term sick leave on grounds of other illnesses (following a car accident, gynaecological operation, etc.), only long-term sick leave as a result of work-related musculoskeletal disorders was taken into consideration. There is currently no standard definition of which illnesses count as work-related musculoskeletal disorders. In the literature, disorders of the back and the upper extremity are considered work-related musculoskeletal disorders [28]-[30].

 ➢ **Long-term sick leave (>42 days) due to psychological impairments to well-being**

Psychological impairments to well-being (burnout and depression) are frequently connected with the emergence of MSD and their progression into chronic conditions [31]-[33]. The Nurse-WIS also includes questions on psychosocial factors, so long-term sick leave because of psychological impairments to well-being has therefore been included in the outcome variable.

 ➢ **Pension for reduced work capacity and/or application for early retirement because of health problems**

Persons who receive a full or half pension for reduced work capacity or who stated that they had applied for early retirement for health reasons were also included in the outcome variable.

### 2.5 Determining the sensitivity, specificity and likelihood ratios of the Nurse-WIS

The sensitivity and specificity of the scale were tested on the basis of the outcome variable (long-term sick leave because of musculoskeletal disorders (MSD) and psychological impairments to well-being, pension for reduced work capacity at T2). A further measure by which the quality of the test can be judged and that takes both sensitivity and specificity into account are likelihood ratios (LR). These are defined as the ratio of proportions of a test result between the sick and the healthy. With dichotomous test results, there is a positive likelihood ratio and a negative likelihood ratio, which are calculated as follows [34]:

$$\mathrm{Positive}\;\mathrm{likelihood}\;\mathrm{ratio}\;\left(\mathrm{LR}+\right)$$

$$\mathrm{LR}+=\frac{\mathrm{Sensitivity}}{1–\mathrm{Specificity}}$$

$$\mathrm{Negative}\;\mathrm{likelihood}\;\mathrm{ratio}\;\left(\mathrm{LR}-\right)$$

$$\mathrm{LR}-=\frac{1-\mathrm{Sensitivity}}{\mathrm{Specificity}}$$

As shown in Table 1, the quality of a test can be classified roughly on the basis of LR+ and LR− [34].

**Table 1:** Rough classification of the efficiency of a screening test or predictive instrument

**LR+**	**LR−**	**Efficiency**
> 10	< 0.1	Very good
5–10	0.1–0.2	Good
2–5	0.2–0.5	Moderate
1–2	0.5–1.0	Poor

### 2.6 The receiver operating characteristic curve (ROC)

The ROC curve is used to show the interaction of sensitivity and specificity by plotting sensitivity against the complementary value of specificity to one (sensitivity versus 1-specificity) in a diagram. The area under curve (ROC AUC) gives an indication of the quality of the test, showing values between 0 and 1. As a rule, values from 0.5 to 0.7 are interpreted as low precision, 0.7 to 0.9 as moderate and a result above 0.9 as high accuracy. The Youden Index (J) was also used to test whether the cut-off value is still valid. J is defined as the maximum vertical distance between the ROC curve and the diagonals and is determined according to the formula *J* = *maximum*(*sensitivity* + *specificity* − 1) [35]. J can reach values between −1 and +1. The closer J is to +1, the better the test is able to distinguish between the sick and the healthy.

### 2.7 Testing the predictive values

The hypothesis is that participants who showed an increased risk according to the Nurse-WIS in T1 will be more likely to take long-term sick leave in the follow-up. A long-term sick leave at T1 as well as older age, gender, education and other factors might lead to the confounding effect that not the Nurse-WIS is the predictor for a long-term incapability to work but one or more of these factors. The influence of these variables and predictors on the predictive value of the Nurse-WIS was also examined. First, bivariate analyses were applied. This was followed by a multivariate analysis based on binary logistic regression. The target variable was the dichotomous outcome variable ‘long-term sick leave or pension for reduced work capacity’. To build a model, the ‘stepwise backwards’ procedure of Hosmer & Lemeshow [36] was applied. The variables that have an influence on the target variable remain in the final model. If the Nurse-WIS prediction for the outcome variable is not based on the influence of other predictors or confounders, the Nurse-WIS variable is expected to be contained in the final model.

## 3 Results

### 3.1 Description of the study population

**Figure 1:**
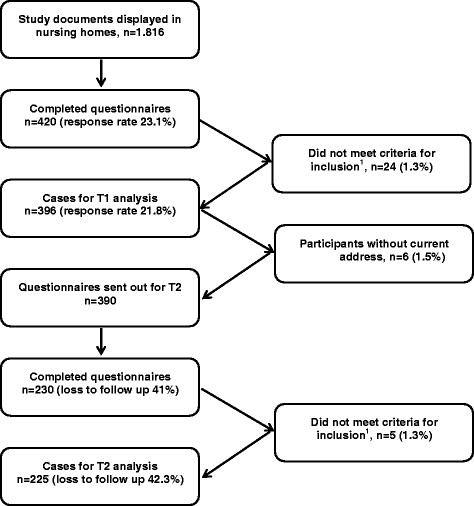
**Flow chart showing the study population for T1 and T2.**^1^Criteria for inclusion: engaged in work as a health care worker (persons on parental leave, voluntary assistants and unemployed persons were excluded), information on periods of sick leave.

T1 consists of 396 study participants (21.8% response). For T2, all 396 persons were addressed. Six study documents were returned, because the addresses were no longer current. For T2, the data from 225 persons (42.3% loss to follow up) was analysed (Figure 1).

The variables for describing the study population of T1 and T2 correlate well (Table 2). At the times of both surveys, the majority of study participants were female, between 36 and 55 years old and more than 60% had completed a three-year training course in geriatric nursing or nursing. Sick leave because of other illnesses was most frequent in both T1 and T2. In both surveys, the proportion of musculoskeletal disorders (MSD) was approximately 20%, while that of psychological impairments to well-being was 6.1% at the time of T1 and 11.6% in T2. As regards cases of long-term sick leave, there was a difference between T1 and T2: the percentage of long-term sick leave cases as a result of MSD was higher in T2, at 6.7%, than in T1, when it was 2.5%. Cases of long-term sick leave due to psychological impairments to well-being were also somewhat more frequent in T2 (2.7%) than in T1 (1.3%).

**Table 2:** Description of the study population

**Variables**	**T1 n = 396**	**T2 n = 225**
	**% (n)**	**% (n)**
**Gender**		
Female	82.6% (327)	86.2% (194)
Male	17.4% (69)	13.8% (31)
**Age**		
17 to 35 years	37.9% (150)	31.1% (70)
36 to 45 years	26.8% (106)	28.4% (64)
46 to 55 years	26.3% (104)	32.0% (72)
Over 55 years	9.1% (36)	8.4% (19)
**Grew up in**		
Germany	86.6% (343)	88.9% (200)
Other countries	13.4% (53)	11.1% (25)
**Education**		
Lower secondary, elementary school certificate	28.5% (113)	28.9% (65)
Secondary school certificate	53.3% (211)	51.1% (115)
High school/university entrance certificate	18.2% (72)	20.0% (45)
**Vocational training**		
Qualified geriatric nurse or nurse	61.9% (245)	64.9% (146)
Geriatric care or nursing assistant	23.7% (94)	23.1% (52)
Employee without nursing training	14.4% (57)	12.0% (27)
**Length of service**		
0–10 years	44.4% (176)	39.6% (89)
11–20 years	30.6% (121)	32.0% (72)
21–30 years	14.6% (58)	16.0% (36)
More than 30 years	10.4% (41)	12.4% (28)
**Scope of employment**		
Full time (≥35 hours a week)	68.9% (273)	69.3% (156)
Part time (15–34 hours a week)	29.3% (116)	29.3% (66)
Part time (<15 hours a week)	1.8% (7)	1.3% (3)
**Working hours**		
Rotating shifts excluding nights	56.6% (224)	57.3% (129)
Rotating shifts including nights	26.3% (104)	23.1% (52)
Day duty, always at the same times	9.8% (39)	11.1% (25)
Only night work	73% (29)	8.4% (19)
**Sick leave (at least 1 day) due to**		
Musculoskeletal disorders	20.5% (81)	20.9% (47)
Psychological impairments to well-being	6.3% (25)	11.6% (26)
Other illnesses^1^	55.1% (218)	39.6% (89)
**Long-term sick leave (>42 days) due to**		
Musculoskeletal disorders	2.5% (10)	6.7% (15)
Psychological impairments to well-being	1.3% (5)	2.7% (6)
Other illnesses^1^	4.5% (18)	4.0% (9)
**Pension for reduced work capacity**		
Application for early retirement because of health problems^2^	0.5% (2)	0.9% (2)
Pension for reduced work capacity	0.0% (0)	0.4% (1)

^1^e.g. accident injuries, acute illnesses (e.g. respiratory or gastrointestinal disorders), gynaecological disorders, degenerative diseases (e.g. arthritis).

^2^One application in T1 led to a (half) pension for reduced work capacity in T2. The other person in T1 did not reply.

### 3.2 Sensitivity and specificity, likelihood ratios and the receiver operating characteristic curve (ROC)

At T1, 28.4% of the 225 individuals who were followed up had an increased risk according to the Nurse-WIS. In the follow-up at T2, 10.2% matched the definition of the outcome variable. This proportion related to 6.7% with a long-term sick leave because of an MSD, 2.7% because of psychological impairments to well-being and 0.9% with a pension for reduced work capacity or an application for a pension. The sensitivity of the Nurse-WIS is 73.9% (95% CI 55.7%–92.3%) and the LR+ is 3.17, so the scale therefore achieves moderate efficiency.

Specificity is 76.7% (95% CI 71.2%–82.8%) and the LR− is 0.34, which also accords with moderate efficiency (Table 3).

**Table 3:** Sensitivity, specificity and likelihood ratios of the Nurse-WIS (n = 225)

	**Total % (n) **	**Outcome variable: long-term sick leave or pension for reduced work capacity**^**1**^**in T2 % (n)**		**LR+**	**LR−**
**Nurse-WIS in T1**		**Yes**	**No**		
		**10.2% (23)**	**89.2% (202)**		
**Increased risk**	28.4% (64)	73.9%^a^ (17)	23.3% (47)	3.17	
**Low/moderate risk**	71.6% (161)	26.1% (6)	76.7%^b^ (155)		0.34
**Total**	100% (225)	100% (23)	100% (202)		

^1^Long-term sick leave because of MSD or psychological impairments to well-being (e.g. burnout), pension for reduced work capacity or application for pension for reduced work capacity.

^a^Sensitivity, Pearson’s chi-square^2^: p-value <0.001.

^b^Specificity, Pearson’s chi-square^2^: p-value <0.001.

LR+ *=* positive likelihood ratio.

LR− *=* negative likelihood ratio.

**Figure 2:**
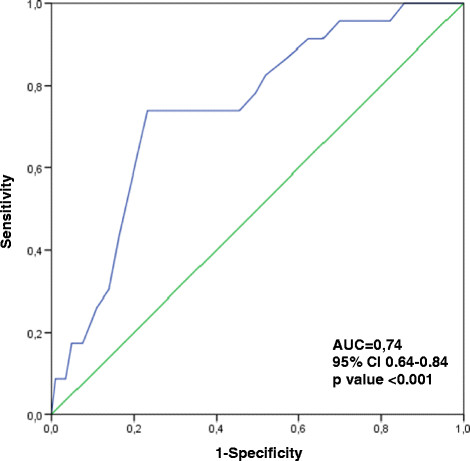
**Receiver operating characteristic curve (ROC).** Blue line = ROC curve. Green line = diagonal. AUC = area under curve.

Figure 2 shows that the ROC curve runs far above the diagonals. The area under curve (AUC) is shown with a significant value of 0.74 (95% CI 0.64–0.84), so the precision of the scale can be described as moderate. The peak of curve is exactly on the values that result for calculating sensitivity and specificity. According to *J*, it can likewise be seen that the highest value is shown at a cut-off of 19.5 points on the total Nurse-WIS score.

### 3.3 Predictive values of the Nurse-WIS

Table 4 shows that women are more likely to have long-term sick leave or a pension for reduced work capacity than men. Also Persons over the age of 55 are more likely to have long-term sick leave or a pension than younger persons. The proportion of persons with long-term sick leave or a pension for reduced work capacity also increases slightly with the length of service. Part-time workers are more often affected than full-time staff and there are also differences as regards working hours. No connection is apparent in the case of persons with other illnesses at T1. However, there is a clear difference in the case of persons who, at T1, had already indicated long-term sick leave (because of an MSD or psychological impairments to well-being) or who had applied for a pension for reduced work capacity. At T2, 66.6% of these were affected by long-term sick leave or were drawing a pension for reduced work capacity.

**Table 4:** Study population, predictive values of the Nurse-WIS and results of the final logistic regression model to test further predictors (n = 225)

**Variables**	**Outcome variable: long-term sick leave or pension for reduced work capacity**^**1**^**in T2**		p value*	**Final model**
	**No % (n)**	**Yes % (n)**		**OR (95% CI)**
**Gender**				
Female	88.7% (172)	11.3% (22)		
Male	96.8% (30)	3.2% (1)	0.166	-
**Age**				
≤ 35 years	88.6% (62)	11.4% (8)		
36 to 45 years	92.2% (59)	7.8% (5)		
46 to 55 years	91.7% (66)	8.3% (6)		
> 55 years	78.9% (15)	21.1% (4)	0.358	**-**
**Education**				
Lower secondary, elementary school certificate	87.7% (57)	12.3% (8)		
Secondary school certificate	88.7% (102)	11.3% (13)		
High school/university entrance certificate	95.6% (43)	4.4% (2)	0.351	-
**Vocational training**				
Qualified geriatric nurse or nurse	90.4% (132)	9.6% (14)		
Geriatric care or nursing assistant	86.5% (45)	13.5% (7)		
Employee without nursing training	92.6% (25)	7.4% (2)	0.640	**-**
**Length of service**				
0–10 years	91.0% (81)	9.0% (8)		
11–20 years	91.7% (66)	8.3% (6)		
21–30 years	86.1% (31)	13.9% (5)		
More than 30 years	85.7% (24)	14.3% (4)	0.692	**-**
**Scope of employment**				
Full time (≥35 hours a week)	92.3% (144)	7.7% (12)		
Part time (<34 hours a week)	66.7% (58)	15.9% (11)	0.060	-
**Working hours**				
Rotating shifts excluding nights/				
Day duty, always at the same times	88.3% (136)	11.6% (18)		
Rotating shifts including nights/				**-**
Only night work	93.0% (66)	7.0% (5)	0.183	
**Long-term sick leave because of other illnesses**^**2**^**in T1**				
No	90.9% (80)	9.1% (8)		
Yes	89.1% (122)	10.9% (15)	0.653	-
**Long-term sick leave or application for pension for reduced work capacity**^**3**^**in T1**				
No	92.1% (199)	7.9% (17)		1
Yes	33.3% (3)	66.7% (6)	<0.001	17.4 (3.34–90.55)
**Predictive values of Nurse-WIS**				
Low/moderate risk	96.3% (155)^§^	3.6% (6)		1
Increased risk	73.4% (47)	26.6% (17)^#^	<0.001	8.2 (2.90–23.07)

^1^Long-term sick leave due to MSD or psychological impairments to well-being (e.g. burnout), pension for reduced work capacity or application for a pension for reduced work capacity in T2.

^2^e.g. accident injuries, acute illnesses (e.g. respiratory or gastrointestinal disorders), gynaecological disorders, degenerative diseases (e.g. arthritis).

^3^Long-term sick leave due to MSD or psychological impairments to well-being (e.g. burnout) or application for a pension for reduced work capacity in T1.

^§^Negative predictive value (NPV).

^#^Positive predictive value (PPV).

*Pearson’s chi-square^2^.

OR = Odds ratio.

95% CI = 95% confidence interval.

The positive predictive value (PPV) of the Nurse-WIS is 26.6%. This means that 26.6% of the persons who, at T1, had indicated an increased risk according to the Nurse-WIS had indeed taken long-term sick leave or drawn a pension for reduced work capacity at T2. The negative predictive value (NPV) is 96.3%. Multivariate analysis shows that persons with an increased risk according to the Nurse-WIS were eight times more likely (OR 8.2, 95% CI 2.90–23.07) to have taken long-term sick leave or drawn a pension for reduced work capacity at T2. Furthermore, persons who had already indicated long-term sick leave or an application for a pension for reduced work capacity at T1 had a 17 times higher risk (OR 17.4, 95% CI 3.34–90.55) of taking long-term sick leave or drawing a pension for reduced work capacity at T2.

## 4 Discussion

In view of the available results it can be summarized that the German version of the Nurse-WIS presents itself as a reliable and valid instrument [24]. The present study is the first prospective study to examine the predictive capabilities of the scale as a follow-up. The predictive values were tested on a collective of 225 geriatric care workers. The Nurse-WIS appears to be able to record an impending period of long-term sick leave or a pension for reduced work capacity. Therefore, it can be assumed that the scale fulfills all requirements to be applied effectively and sensible as a screening instrument, but some implications for the practical use of the Nurse-WIS should receive attention. In the results of the baseline survey it was shown that the German version of the Nurse-WIS meets the measurement requirements as defined by modern psychometric theory [24]. The German version of the Nurse-WIS is based on the original English version of the Nurse-WIS developed by Gilworth et al. [23], which also has good psychometric characteristics.

### 4.1 Special features of the study design and representativeness of the random sample

Within the framework of the study to validate the Nurse-WIS, a cohort of geriatric care workers were surveyed prospectively over twelve months at two survey times (baseline T1, follow-up T2) on the basis of a questionnaire. At T1, a response rate of 23.1% was achieved.

Because of the method of recruitment of the study participants it is possible that the response rate for T1 is somewhat underestimated (more information about the recruitment and the special features of the study design can be seen at [24]).

This seems probable because at T2, a considerably higher percentage of participants (57.7%) answered the second questionnaire. This time, study participants who had signed a declaration of consent (and given their addresses) had a questionnaire sent to their home. This is significant especially because participants in follow-up surveys are asked a second time to fill in a questionnaire and, as a rule, one tends to expect a lower percentage of participants who send back their questionnaire than in the baseline survey.

Regardless to the relatively high percentage of participants who answered the second questionnaire in T2 compared to T1, the loss to Follow-up was at least 42.3%. However, this might lead to a selection bias, e.g. if more health care workers affected by musculoskeletal disorders tend to answer the questionnaire than their healthier counterparts. We conducted a non-responder analysis to assess the impact of the loss to follow up and it showed that the health care workers who did not participate in T2 are more likely to be male, younger and that they have a slightly lower risk according to the Nurse-WIS. But there was no difference between participants and non-responders referring to sick leave or long-term sick leave because of MSD or Psychological impairments to well-being at T1.

At both survey times, more than 80% of the study participants were female. As can be confirmed from information provided by the German Institute for Employment Research (Institut für Arbeitsmarkt und Berufsforschung (IAB)), the proportion of women in social care occupations, which includes geriatric nursing in Germany, is also 80%. Based on their data, the age distribution in the present study is also comparable with the age distribution in social care occupations in Germany [37].

At both T1 and T2, around 20% stated that they were unable to work because of a musculoskeletal disorder (MSD) in the preceding 12 months. The literature suggests somewhat higher annual prevalence rates (40 to 50%) of low back pain in nursing staff. However, those studies do not record sick leave because of MSD but rather the symptoms of an MSD such as back pain or neck pain [7],[38]-[42]; yet pain does not necessarily lead to health care workers staying away from work and taking sick leave.

After MSD, psychological impairments to well-being were the most frequent reason for sick leave, with the proportion increasing slightly from T1 (6.3%) to T2 (11.6%). The literature also shows that nursing staff are affected by stress reactions such as burnout and psychological problems [12],[14]-[16],[43]. In the case of long-term sick leave, there is a difference between T1 and T2. Long-term sick leave because of MSD is more frequent at T2, at 6.7%, than at T1, when it was 2.5%. This may indicate that nurses who were already suffering from MSDs were more likely to have taken part in this survey.

Apart from that, one can conclude that the random survey in the present study is comparable with other studies. However, the relatively low response rate at T1 and the relatively high loss to follow up rate at T2 should be considered.

### 4.2 Predictive capability and implications for the practical use of the Nurse-WIS

The reliability and validity of the German version of the Nurse-WIS were assessed by various methods, during the course of which a German scale consisting of 28 items in total was established [24].

The values for the prognostic validity of the Nurse-WIS are moderate and overall one can therefore assume that the Nurse-WIS is able to predict an impending long-term sick leave or the drawing of a pension for reduced in work capacity. Sensitivity, the measure by which the Nurse-WIS correctly identified the proportion of persons with a long-term sick leave or a pension for reduced work capacity one year in advance, is around 74%. Specificity is around 77%. When the research group around Gilworth et al. [23] was developing the Nurse-WIS, they found similar sensitivity values of 75% but achieved a considerably higher value of 100% for specificity. However, in order to establish sensitivity and specificity, Gilworth et al. [23] arranged for some of the study participants (n = 27) to be examined individually by an occupational therapist using a standard protocol. In the present study, information on sick leave and pensions for reduced work capacity that had been provided by study participants themselves was taken from the questionnaires in order to determine these values. A personal assessment of a study participant by an occupational therapist may be more reliable, but measures something other than long-term sick leave. That may explain the divergent value for specificity. The occupational therapist only recorded the status of work instability without testing whether long-term sick leave occurred or a pension for reduced work capacity was paid at a later date. There was therefore no prospective examination. Yet work instability diagnosed by an occupational therapist does not necessarily lead to long-term sick leave or a pension for reduced work capacity. This prospective investigation was carried out for the first time in the present study and moderate values for sensitivity and specificity were found at a cut-off value of 20 points.

By changing the cut-off value it would theoretically be possible to vary the sensitivity and specificity. For example, by reducing the cut-off value to 10 points, one could achieve a higher sensitivity of more than 87%. In that case, however, specificity would be considerably lower at only 37%. Higher sensitivity can therefore only be bought at the cost of lower specificity and vice versa [34],[44]. Moreover, it would not make sense to change the original cut-off value of 20 points for the German version of the Nurse-WIS, since analysis using the ROC curve and the Youden Index confirmed that the highest precision is achieved with this cut-off value.

Further important values for a potential screening instrument are the predictive values. The negative predictive value (NPV), i.e. the proportion of persons at no risk according to the Nurse-WIS and who were actually healthy one year later, is high at 96.3%. In the case of the positive predictive value (PPV) it was found that around one quarter of persons with an increased risk according to the Nurse-WIS subsequently had a period of long-term sick leave or drew a pension for reduced work capacity. This connection also remained significant in the multivariate analysis, which showed that the probability of taking long-term sick leave or of drawing a pension for reduced work capacity is around eight times higher for persons with an increased risk according to the Nurse-WIS. Nevertheless, the PPV initially appears relatively low. However, it should be taken into account that for a screening, the predictive values always depend on the prevalence of illness. If prevalence is low, the positive predictive value is also low, even in the event of tests with a good efficiency and a high sensitivity and specificity [34],[44]. In the follow-up survey, the prevalence of long-term sick leave or drawing of a pension for reduced work capacity was around 10%. According to Bender [34], the predictive values for screening tests to be expected according to prevalence can be read off a table if the sensitivity, specificity and test efficiency are known. Accordingly, if prevalence is 10%, for tests with a sensitivity and specificity of around 70% and moderate test efficiency a value of around 21% is indicated. This value is roughly comparable with the PPV of 27% in the present study. According to Bender [34], if prevalence is increased to 50%, for example, a PPV of around 70% can be expected.

Consequently, in clinical practice, when applying a screening tool it is desirable to do so in populations with a high prevalence and/or an increased risk of illness [34],[44].

In the present survey, the predictive value of the Nurse-WIS was determined for the period of 12 months after the survey. Assuming that the prevalence of long-term sick leave and pensions for reduced work capacity rises as the period of observation increases, it is likely that the PPV will improve if a longer follow-up period of 24 months is chosen, for example.

In order to improve the PPV it would also be conceivable to use the Nurse-WIS primarily for health care workers who show the first signs of an MSD but have not yet sought medical help. This could be done by means of an entry criterion, i.e. by supplementing the Nurse-WIS with a preliminary question and asking only those who reported significant musculoskeletal symptoms (lasting more than two hours at a time) in the previous three months to complete the Nurse-WIS.

It would also be conceivable to use the Nurse-WIS primarily for health care workers over 50 years of age. This seems reasonable considering an increased occurrence of long-term sick leave and pensions for reduced earning capacity in people aged 50 or older. The presence of a long-term sick leave as well as older age, gender, education and other factors might lead to the confounding effect that not the Nurse-WIS is the predictor for a long-term incapability to work but one or more of these factors. We conducted a multivariate analysis based on a binary logistic regression to check for these confounding effects. It was shown that the Nurse-WIS was contained in the final model and that means that the prediction of the Nurse-WIS is not based on the influence of other predictors or confounders. Along with the Nurse-WIS the presence of a long-term absence from work due to MSD or a psychological indisposition a year before the follow up proved to be a significant predictor for a long-term incapability to work or pensions for reduced earning capacity. It can therefore be assumed, that if the surveyed group is restricted to health care workers with a particular risk (e.g. older than 50 years, first signs of MSD) the PPV of the Nurse-WIS will improve. However, further research is needed to test these hypotheses.

### 4.3 Use of the Nurse-WIS to maintain the working capacity of nursing staff

In order to counter the forecast lack of health care workers due to demographic change, the importance of maintaining the working capacity of nursing staff is becoming a central issue. A systematic review of 63 studies on intervention strategies to reduce musculoskeletal injuries associated with handling patients showed that multifactor interventions based on a risk assessment are most likely to succeed [18]. Moreover, research has shown that interventions aimed at secondary prevention and including persons at risk of early retirement or of drawing a pension for reduced work capacity [22], or with the first symptoms of a musculoskeletal disorder [19]-[21], have mainly proven to be effective. It can therefore be assumed that offers of this nature would also be useful for maintaining the working capacity of health care workers. It would be conceivable to use the Nurse-WIS to identify nursing staff at risk and therefore facilitate the use of preventive offers and to design these efficiently. The use of the Nurse-WIS as a management instrument in rehabilitation can also be assumed to be beneficial. The goal of rehabilitation is to maintain, improve or restore the working capacity of people threatened with disability, according to their capability, and if possible to ensure their working capacity in the long term. One particular goal of rehabilitation in Germany is reintegration into working life. As part of an individual, staged plan, the sick, disabled person is introduced step by step or hour by hour to the stresses and strains of the former workplace, until full capacity to work is reached [45]. The German version of the Nurse-WIS could also be helpful in this step-by-step reintegration into working life. It could be used, for example, to check the extent to which the health care worker can be exposed to occupational stresses and strains again. However, when using the Nurse-WIS as a management instrument for health-promotion, prevention or rehabilitation measures, it should be noted that in the present study around one quarter of the geriatric care workers surveyed indicated an increased risk according to the Nurse-WIS and the positive predictive value (PPV) of the scale is around 27%. Because of this it would be useful to use further management instruments alongside the Nurse-WIS, such as an additional examination of health care workers with an increased risk according to the Nurse-WIS by an occupational therapist, company doctor or physician, so as to confirm the findings. Moreover, when using the Nurse-WIS as a management instrument it would be interesting to see the PPV in further studies and among other nursing staff, e.g. hospital workers. There are other more general scales (e.g. WAI, SF-12) with good psychometric properties, but these scales often focus on the health-related quality of life or on disability and function. Currently, as far as we are aware, the Nurse-WIS is the first occupation-specific scale to focus on health care workers experiencing work instability. And since there is evidence that early interventions are more effective, the identification of work instability in health care workers might be helpful to ensure that they have rapid access to these interventions.

## 5 Conclusion

Demographic trends in Germany mean that, among other things, it is important to keep health care workers healthy and motivated to work in their occupation until they reach retirement age. This underscores the importance of prevention and health promotion in order to maintain the working capacity of nursing staff. However, until now occupation-specific screening instruments for identifying health care workers at risk so as to offer targeted prevention measures at an early stage are missing and therefore the validation of the Nurse-Work Instability Scale (Nurse-WIS) was undertaken. Along with the study on the development of the Nurse-WIS [23], this study is the only validation study to date and is also the first study to have tested the prognostic value of the Nurse-WIS by means of a follow-up survey. During this process the German version of the scale was shown to be an easy-to-use, reliable and valid instrument with satisfactory predictive capabilities. However, before using the Nurse-WIS as a management instrument some implications for the practical use of the scale should receive attention (e.g. confirmation of the findings from an occupational therapist or physician). Moreover, further research is needed to confirm these findings and it would be interesting to see the predictive value in further studies and among other staff (e.g. hospital workers) and among staff with a particular risk (e.g. aged over 50, early signs of an MSD).

Overall, the results were very promising, and the use of the scale in research, evaluation and practice could contribute indirectly towards countering the premature departure of health care workers from the workforce and the anticipated shortage of nursing staff.

## Abbreviations

Nurse-WIS: Nurse-Work Instability Scale

MSD: Musculoskeletal disorders

95% CI: 95% confidence interval

LR+: Positive likelihood ratio

LR−: Negative likelihood ratio

OR: Odds ratio

PPV: Positive predictive value

NPV: Negative predictive value

ROC curve: Receiver operating characteristic curve

BGW: German Institution for Statutory Accident Insurance and Prevention in the Health and Welfare Services

CVcare: Competence Centre for Epidemiology and Health Services Research in Nursing

## Competing interests

The authors declare that they have no competing interests.

## Authors’ contributions

MH participated in the design of the study, performed the statistical analysis and drafted the manuscript. AS and CP helped to develop the questionnaire and participated in data collection. AN conceived the study and participated in its design and coordination, and helped to draft the manuscript. All authors have been involved in revising the manuscript critically for important intellectual content and approved the final manuscript.
